# Illustrations of the Efficacy of Compression and Percussion in the Cure of Rheumatism and Sprains, Scrofulous Affections of the Joints and Spine, Chronic Pains Arising from a Scrofulous Taint in the Constitution, Lameness, and Loss of Power in the Hands from Gout, Paralytic Debility of the Extremities, General Derangement of the Nervous System; and in Promoting Digestion, with All the Secretions and Excretions

**Published:** 1824-09

**Authors:** William Balfour

**Affiliations:** the University of Edinburgh.


ART. III.—*Illustrations of the Efficacy of Compression and Percussion in the Cure of Rheumatism and Sprains, Scrofulous Affections of the Joints and Spine, chronic Pains arising from a Scrofulous Taint in the Constitution, Lameness, and Loss of Power in the Hands from Gout, Paralytic Debility of the Extremities, General Derangement of the Nervous System; and in promoting Digestion, with all the Secretions and Excretions.* By WILLIAM BALFOUR, M.D. of the University of Edinburgh.

[Continued from page 115.]

CASE XXXI.—Mrs. D., aged twenty-seven, became my patient on the 30th April, 1822. She was affected with pain in the shoulders and muscles of the neck; along the whole course of the spine, from the occiput to the coccyx inclusive; over all the ribs, particularly at their anterior or cartilaginous ends; in the point of the breast-bone; in the muscles of the loins; along the tops of both haunch-bones; in the hips, in the hip-joints; in the thighs, in the legs. It is almost needless to say that these symptoms were accompanied with the greatest degree of debility. The patient was just capable of locomotion, and that was all. With the aid of a stick grasped firmly in both hands, she could rise from her seat, by an exertion of five minutes' continuance, and then transport herself, leaning on the stick in both hands, to a little distance, by shoving her feet alternately, one inch at a time, along an uncarpetted floor. She could not raise her feet, in any degree, from the floor. She was lifted into and out of bed, like a child. Her knees and feet were permanently fixed, at about two inches distance from each other; she could neither separate them further, nor make them approximate nearer, on

---

account of pain in the hip-joints. She informed me, she was sure the opposite sides of the pelvis had approximated considerably since her last child. She had been in this state for three years and a half, without experiencing any other change than increase of debility and pain. All the remedies, external and internal, which had been prescribed, had no beneficial effect, either in removing or in arresting her complaints.

Before I discovered the efficacy of compression and percussion in rheumatism, and complaints allied to it, I would have considered this case hopeless; and would have got off, like those who preceded me, by prescribing exercise in the open air and sea-bathing. The former, however, the patient could not take; and her condition in life precluded her from availing herself of the latter: she must, therefore, have been for ever lost to her family. Even with all my experience of the efficacy of the new practice, my hopes of effecting a cure were any thing but sanguine. Indeed, had I been required to give a decided opinion, it would have been that her extreme debility, the nature and inveteracy of her complaints, combined with the multiplicity of the parts affected, forbad the hope of her condition ever being much ameliorated. But my opinion was never asked. The reports which had reached the patient of the success of my practice in similar cases, had inspired her with perfect confidence that she also would be benefited by it: she, therefore, resigned herself to my care, without ever entertaining a doubt of a favourable issue.

If the reader can imagine a person so generally affected with disease, and so debilitated, that the slightest touch on any part of the trunk of the body would not only occasion pain, but cause a vibration of the whole nervous system, then he will have some faint idea of the condition of this patient. It may well be supposed, therefore, that I commenced my operations with the utmost gentleness and caution: not that this heroic woman objected to any degree of pain which it might be necessary for her to suffer, but a rude and forcible mode of proceeding would not have accelerated the cure. It is by tact and skill that such cases are successfully managed; and this tact and skill are to be acquired, as every other medical qualification is acquired—from practice.

The affections of the external and soft parts quickly yielded to compression and percussion; not so the cartilaginous and deep-seated. When percussion was applied to the sacrum, however slightly, intolerable pain was produced in all the connexions of the os innominata, and darting through the centre of the pelvis, issued, as it were, from between the crura of the pubis. When the pubis was firmly supported, however, these effects were not produced: on the contrary, percussion could be applied to any part of the pelvis, with the greatest good effect. This course was, therefore, followed. Slight percussion on the inferior lumbar vertebræ caused a nervous vibration to the very toes, which was rather pleasurable than otherwise. But the most formidable difficulties I had to surmount in this case were defined, painful spots all round the brim of the acetabulum, which obstructed the motion of the hip-joints. These points it was necessary for me to reach with my fingers, for the purpose of compressing them, before passive motion of the joints could be attempted. This it was no easy matter to accomplish. I succeeded, however, not only in reaching all the affected points, but also in removing the pain in a few days, with one exception only. This point, which for ten days set all my efforts at defiance, was situated on the front and very flexure of the joint, exterior to the course of the femoral artery. It was extremely minute, and I often reached it, but never in such a way as to remove the pain. Conceiving, therefore, that this was owing to some inequalities, which required a greater degree of compression than I had yet employed, I one day, after putting the thigh in a proper position, pushed the point of my fore-finger as deep into the flexure of the joint, at the pained part, as I could; when, depressing the palm of my hand and raising the point of my finger, which I still kept closely applied to the part, I struck the point I was in search of fairly in front. The patient immediately called out, "You have it now; give it no quarter." The pain was removed in less than half a minute, and never returned. The motion of the hip-joints was now perfectly free, and remained so. Pain was also removed from every point of which she complained; so that, at the end of three weeks from the time I was called in, a complete cure was effected. The patient, however, as may well be supposed, had still to contend with a considerable degree of debility; which time, air, and exercise alone could effectually remedy: but of these she could now avail herself, as her strength had been so far restored by the operations, that she was able to go to the country; and I have not heard of her since.

In the course of my attendance on this patient, she observed one day, that "surely the operation of compression and percussion must have a beneficial influence on the general health, as I find my appetite greatly improved since their commencement: nay, I am compelled to eat immediately after every operation, before I have time to put my dress in order." This observation was spontaneous, and quite accords with that of many others of my patients.*

* The great length of this paper, and the press of other matter, have obliged us to omit some parts of it; among others, Cases 32 and 33: the former an instance of severe rheumatism, and the latter of a tumor on the sole of the foot, cured by the remedy so strongly recommended by the author.—EDITORS.

CASE XXXIV.—A lady, aged sixty, consulted me in April, 1822, for a complaint that excites but little sympathy, and is often made a subject of merriment, but which is nevertheless sufficiently distressing to those who are afflicted with it. She was of a delicate constitution, and had for many years been subject to paroxysms of low spirits, uneasy feelings, and nervous irritation. Unable to take exercise in the open air, she was often so oppressed in her spirits and feelings, that she could neither lie, sit, stand, speak, or listen to others. On such occasions, existence itself was rather a burden than a pleasure.

It was from having heard, from some of her lady acquaintances, of the beneficial effects of my operations on their health and spirits, that this patient applied to me; and, happily, I was not long of convincing her that their reports had not been without foundation. As she had no local pain, I satisfied myself with applying percussion to the spine, shoulders, and superior extremities. This I did gently at first, but gradually increased the force, as far as was agreeable to the patient's feelings. The effects were an improvement of feeling and a buoyancy of spirits, far exceeding her expectations. I explained to her the principles of my practice, which she readily comprehended, and considered the information as a most important acquisition. She had the operation performed by her servant afterwards, as occasion required; by which means, she said, she could command exercise, almost equal to equestrian. The fact is, if equestrian exercise possesses the advantage that the patient enjoys the open air in addition to the simultaneous concussion of the whole body, percussion has this superiority to both gestation and walking, that it may be applied at any time; that it is applicable to the most delicate, without occasioning fatigue; that it may be applied with precisely the requisite degree of force; and so as to produce local or general effects, as the case requires.

I saw this lady some months afterwards, when she informed me she found her health and strength greatly improved by percussion, which had enabled her to take much more exercise in the open air, and that she was now a stranger to those paroxysms of low spirits, uneasy feelings, and nervous irritation, for which she had originally consulted me: at any rate, that she was able to repel their approach at any time, and in half a minute. I met her yesterday, (21st June,) returning from an excursion into the country, walking at the rate of four miles an hour.

CASE XXXV.—Mr. D., aged thirty, of a very fair complexion, with large upper lip, and other signs of a scrofulous diathesis, became my patient in April, 1822. He complained of pain and stiffness of the joint of the lower jaw-bone, which prevented him from opening his mouth sufficiently to receive and chew his food; of pain and stiffness of his neck, which rendered his head immovable in any direction; of pain and rigidity of the shoulders; of pain along the whole course of the spine, which was considerably bent forward, and gave the patient a stooping gait; of pain in the point of the breast-bone, and cartilages of all the false ribs; in the loins; all along the tops of both haunch-bones; in the hips, where the muscles were knotted in a surprising degree; in the tendons of both heels, particularly at their insertion. These complaints were of nine years' standing, and had reduced the patient to a great degree of debility. When I was called, he took three-quarters of an hour to walk half a mile, and that with the greatest constraint and pain.

If it is an established doctrine in pathology, that parts which, in a sound state, are possessed of little sensibility, become sensible when affected with disease, in an inverse ratio; then, simply to say that this patient was pained in such and such places, is to convey but a very faint idea of the actual facts of the case. Many are pained in the same regions, but not in the same structures, and therefore do not suffer the twentieth part of the pain which he endured. His complaints were not confined to the muscles or soft parts, though these were very considerably affected: they were deep-seated in the immediate coverings of the bones and cartilages, if not in the very substance of the cartilages themselves. These circumstances, equally distressing to the patient and formidable to the practitioner, rendered a cure infinitely more difficult than if the soft parts had been exclusively affected.

I applied compression above and below the zygomatic arch, as directly on the maxillary hinge as possible, with good effect;* and then percussion to the chin, while the patient kept the strong muscles which elevate the lower jaw in as relaxed a state as possible. By these means, I succeeded in enabling the patient to open his mouth to a very considerable extent, at every operation, and ultimately to open and shut it without foreign aid.

In order to give motion to the head, I insinuated my fingers among the muscles of the neck as deeply as possible, that I might compress the cartilages and their coverings which belong

* "A short time after I had been in attendance, a singular circumstance occurred, which afforded me an opportunity of demonstrating, to a gentleman of eminence in the profession, the efficacy of the mode of treatment I had adopted in regard to his lordship.

"A small, but extremely painful, tumor suddenly appeared on the ramus of the lower jaw, right side, immediately under the zygomatic arch. It impeded greatly the motion of the jaw, but disappeared almost entirely upon the application of simple pressure. It recurred, however, for three or four days successively; and, on one occasion, shut almost entirely the patient's mouth. The gentleman alluded to was present that day when I called, and I requested him to stop till I should remove the tumor, and enable his lordship to open his mouth; both which I expected to accomplish in the course of three minutes. The event justified my expectations, in half the time specified."—See my *Treatise on Rheumatism*, Case 30, page 217; 1819.

to the vertebræ, where chiefly the mischief lay. This it was no easy matter to accomplish, considering the acute sensibility of the parts affected; but *omnia vincit labor*. These efforts I alternated with passive motion of the head, which was effected by making the patient shut his mouth, and then applying percussion, first under the chin, and then contrariwise. This produced motion of the head, backwards and forwards. I gave lateral motion, by fixing the neck with one hand, grasping the crown of the head in my other, and giving it (the head) a slight jerk, first to one side, and then to the other. Rotatory motion was effected, by taking the chin in one hand, (the patient keeping his mouth closely shut,) and the upper and back part of the head in the other, and then giving it (the head) a slight rotatory jerk, which was equivalent to both compression and percussion on the very points obstructing motion.

For a considerable time, little or no impression seemed to be made on the disease, the nature of the parts concerned (spinal marrow) precluding the idea of force. Skill, tact, and perseverance alone, were here available; and a most important matter it is, that any thing is of avail in such a case. In little more than four months, pain was removed from the neck, motion given to the jaw, and to the head in every direction.

The other parts could be treated with more freedom, and, though obstinate, also gave way within the time specified. The knots in the glutei muscles were extremely tender, but disappeared under compression, as completely and permanently as if they had never existed. In fine, by the end of summer, the patient could walk like any other person, and was able to keep on his feet for many hours together; a consummation which a great physician, now no more, predicted, long before, would never take place.

Case XXXVI.—Mr. A., aged thirty-six, a jockey, consulted me, in April 1822, for lameness in both feet; but, whether occasioned by gout or rheumatism, I leave the reader to form his own opinion. In other respects, the man was in perfect health, full of flesh and blood, and lived rather freely. Both feet were much swollen, from the ankle-bones to the points of the toes inclusive, and in some parts red and shining. There was great pain at the base of the inner ankle-bones, in the ankle-joints, among the tarsal bones, and in the soles of the feet, along the course of the tendons of the toes. In walking, which he did with great difficulty and pain, the patient had no spring or motion in the feet themselves, but lifted and put them down perpendicularly, as if they had been mere stumps. He scarcely ever attempted to walk, indeed; as, when he went abroad, it was always on horseback. The disease had existed a great length of time, had become gradually worse, and, when I was called, completely prevented rest in the night-time.

It is with rheumatism as with other diseases: some cases appear trifling at first, but prove obstinate ultimately; while others are formidable at first view, but yield sooner and more easily than could have been justly expected. Of the latter, the present case is an instance. Notwithstanding the parts affected were so acutely pained, that, in the application of compression, and also in giving passive motion to the ankle-joints and metatarsal bones, the utmost caution was requisite; yet this man was only sixteen days under my care, during which he had no more than eight operations, when he found himself independent. Had my opinion been asked at first, I would have said that months would elapse before a cure could be effected. The first symptom of amendment that occurred was perfect rest in the night-time; whereas, he had not, for a great length of time, known what a sound sleep meant. In percussion, gently applied, the patient delighted: it created a sensation rather pleasurable than otherwise, and principally contributed to effect motion of the bones on each other. Without it, no cure of this case could have been effected; because all the usual remedies had had a fair trial, in vain, long before I was consulted.

Case XXXVII.—A gentleman, aged forty, became my patient in May, 1822. He complained of vertigo; muscæ volitantes; frequent sickness, with disposition to vomit; loss of appetite; obstinate costiveness; frequent pain in the bowels, especially in the night-time, "as if torn by vultures;" pervigilium; rheumatism in his loins, thighs, legs, and soles of his feet. The whole nervous system was so debilitated and unhinged, that he could not stand. All his friends thought him dying; and so persuaded was he of this himself, that he had settled his affairs. He had been ill for years, but how long I cannot tell. There was no secret made, however, of his complaints having been induced by free living alone.

Four or five practitioners, of the first eminence, had attended this patient, together or successively; but all had drawn off, without having been able to render any material assistance. It must not be supposed, however, that I intend the slightest reflection on these eminent individuals. I only mean to show that compression and percussion are available in many cases and circumstances, in which medicine has little or no power. I put it to the common sense of mankind, which of the modes of cure is preferable,—that which was prosecuted for years, before the patient's strength was greatly reduced, without any beneficial result; or that which in a few weeks restored him to health and strength, after having been brought to the brink of the grave? I put it to the common sense of mankind, if the discovery of so efficient a remedy as compression and percussion proved in this instance, is not of the highest practical importance?

When I commenced my treatment of this case, I found it necessary to proceed with as much caution as if an infant had been under my hands. This was owing to the extreme sensibility of the nervous system, which was so susceptible, that a very slight degree of percussion on the spine caused a vibration through the whole frame. At first, this operation occasionally produced a slight and momentary degree of sickness; and the patient soon after vomited a mouthful of an offensively sour, greenish substance, and immediately felt greatly relieved. As the nervous system acquired tone, however, these effects disappeared, and, instead of sickness being produced, the stomach was invigorated, and the appetite improved. Not that the sickness and vomiting, which occasionally took place when the operations were first employed, were to be regarded as hurtful; on the contrary, they were the most salutary: they were the effects of a gentle stimulus communicated to the stomach, by which it was enabled to throw off offensive matter.

I found my patient had been in the habit of swallowing great quantities of aloetic pills, without reflecting that, where the ingesta are small, the egesta cannot be copious. I had no difficulty, however, in convincing him of the impropriety of such a proceeding; and that the racking pains in his bowels were partly attributable to the drying and heating medicine, which he took in such enormous doses, and so frequently. Accordingly, the pills were instantly given up; and aleberry, for supper, was resorted to with good effect. Gentle compression and percussion were also applied to the abdomen, as well as to the spine, every day, with deliberation and perseverance.

The rheumatic affection of the loins and inferior extremities was very severe, and, consequently, required the most delicate management. At first, compression, not greater than sufficient to break an egg-shell, could scarcely be borne; and a very slight degree of percussion affected the whole frame precisely like a shock of electricity. I was no way intimidated, however, by these circumstances; because I believed, and assured my patient, that every operation would diminish the extreme sensibility of the parts, and of the system at large. I was not mistaken. For a while, indeed, little or no impression seemed to be made on the complaint; but, at the end of about three weeks, general amendment was evident. The vertigo and muscæ volitantes now recurred less frequently, and were experienced more slightly; the stomach and bowels had considerably recovered their tone and functions;* the limbs their strength; sleep had returned; and the whole nervous system was invigorated. Compression and percussion, the only remedies employed from beginning to end in this case, could now be applied with

* In my preliminary observations, reference was made, by mistake, to Case 40, instead of this, as an instance of "intestinal excretion being greatly promoted by percussion to the abdomen."

considerable freedom; not only without inconvenience to the patient, but with immediate and insensible improvement of feeling. On these data, I considered a cure as now certain, and that perseverance alone was wanting to complete it. Soon after this, the patient was able to rise from a chair, to stand, and sit down, without aid. Then he came to walk through his room; then to walk down and up stairs, without help; then to walk in front of the house; then to go to the country in his carriage, and take a walk on a country road; and, at the end of seven weeks from the time I was called, I left him free from complaint, and walking over all the town.

*Summary.*—Case of a gentleman, for years afflicted with a nervous affection of the head; indigestion, sickness, and vomiting; pains in the bowels, as if torn by vultures; costiveness; rheumatism, from the loins to the soles of the feet inclusive; he could neither walk, nor stand, nor rise to his feet, the whole nervous system being in a state of great debility and derangement. Cured, so as to be able to walk all over the town, in seven weeks.

[To be continued.]

